# Fully automated and highly specific plasma β-amyloid immunoassays predict β-amyloid status defined by amyloid positron emission tomography with high accuracy

**DOI:** 10.1186/s13195-022-01029-0

**Published:** 2022-06-23

**Authors:** Kazuto Yamashita, Masahiro Miura, Shunsuke Watanabe, Kengo Ishiki, Yuji Arimatsu, Junko Kawahira, Toshiko Kubo, Katsutaka Sasaki, Takayuki Arai, Kei Hagino, Yasuhiro Irino, Kota Nagai, David Verbel, Akihiko Koyama, Shobha Dhadda, Hayato Niiro, Shigeki Iwanaga, Toshiyuki Sato, Tomokazu Yoshida, Atsushi Iwata

**Affiliations:** 1 Central Research Laboratories, Sysmex Corporation, Kobe, Japan; 2 Business Incubation Division, Sysmex Corporation, Kobe, Japan; 3 Sysmex R&D Center Americas, Inc., Mundelein, IL USA; 4 Reagent Engineering Division, Sysmex Corporation, Kobe, Japan; 5 Japan and Asia Clinical Development Department, Eisai Co., Ltd., Tokyo, Japan; 6 Biostatistics, Eisai Inc., Nutley, NJ USA; 7 Translational Science, Eisai Inc., Nutley, NJ USA; 8 Biostatistics and Project Operations, Eisai Inc., Nutley, NJ USA; 9 Medical Affairs Division, Sysmex Corporation, Kobe, Japan; 10 Sysmex Corporation, Kobe, Japan; 11 Department of Neurology, Tokyo Metropolitan Geriatric Hospital, Tokyo, Japan

**Keywords:** Alzheimer’s disease, Beta-amyloid, Biomarker, Diagnosis, Immunoassay, Plasma, Aβ42/Aβ40

## Abstract

**Background:**

Clinicians, researchers, and patients alike would greatly benefit from more accessible and inexpensive biomarkers for neural β-amyloid (Aβ). We aimed to assess the performance of fully automated plasma Aβ immunoassays, which correlate significantly with immunoprecipitation mass spectrometry assays, in predicting brain Aβ status as determined by visual read assessment of amyloid positron emission tomography (PET).

**Methods:**

The plasma Aβ42/Aβ40 ratio was measured using a fully automated immunoassay platform (HISCL series) in two clinical studies (discovery and validation studies). The discovery and validation sample sets were retrospectively and randomly selected from participants with early Alzheimer’s disease (AD) identified during screening for the elenbecestat Phase 3 program.

**Results:**

We included 197 participants in the discovery study (mean [SD] age 71.1 [8.5] years; 112 females) and 200 in the validation study (age 70.8 [7.9] years; 99 females). The plasma Aβ42/Aβ40 ratio predicted amyloid PET visual read status with areas under the receiver operating characteristic curves of 0.941 (95% confidence interval [CI] 0.910–0.973) and 0.868 (95% CI 0.816–0.920) in the discovery and validation studies, respectively. In the discovery study, a cutoff value of 0.102 was determined based on maximizing the Youden Index, and the sensitivity and specificity were calculated to be 96.0% (95% CI 90.1–98.9%) and 83.5% (95% CI 74.6–90.3%), respectively. Using the same cutoff value, the sensitivity and specificity in the validation study were calculated to be 88.0% (95% CI 80.0–93.6%) and 72.0% (95% CI 62.1–80.5%), respectively.

**Conclusions:**

The plasma Aβ42/Aβ40 ratio measured using the HISCL series achieved high accuracy in predicting amyloid PET status. Since our blood-based immunoassay system is less invasive and more accessible than amyloid PET and cerebrospinal fluid testing, it may contribute to the diagnosis of AD in routine clinical practice.

**Supplementary Information:**

The online version contains supplementary material available at 10.1186/s13195-022-01029-0.

## Introduction

Alzheimer’s disease (AD) is the most common neurodegenerative disease worldwide [1]. It places a huge burden on patients, their caregivers, and society [2, 3]. The first pathological change in AD is the accumulation of β-amyloid peptide (Aβ) as amyloid plaques in the brain, beginning 15–25 years before symptom onset [4–6].

Many disease-modifying therapies (DMTs) targeting Aβ have been studied as treatment for AD [7–9]. Recently, the US Food and Drug Administration approved aducanumab (Aduhelm) for patients with early AD (mild cognitive impairment [MCI] due to AD and mild AD) using their accelerated approval pathway, based on its reduction of amyloid plaques in the brain, making it the first potential DMT for AD to be available for clinical use in the USA [10]. According to the prescribing information for aducanumab, there is no specific requirement to confirm Aβ-positivity prior to treatment. However, the appropriate use recommendations published by an expert panel suggested that it is necessary to confirm Aβ-positivity prior to initiating the treatment [11, 12]. In the clinical trials of aducanumab, amyloid positron emission tomography (PET) was used to confirm the brain Aβ status [13]. The expert panel suggested that the assessment of cerebrospinal fluid (CSF) biomarkers (Aβ, total tau, and phosphorylated tau) may be an alternative to amyloid PET [11]. Moreover, clinical trials of other DMTs for AD treatment have also been required to confirm Aβ status in the brain, and the confirmation of Aβ-positivity is required for the diagnosis of AD [14, 15].

Amyloid PET and CSF testing are widely accepted in research settings for the assessment of Aβ status. However, their use is burdensome in routine clinical practice, given issues with their accessibility, cost, and invasiveness [16, 17]. Blood-based biomarkers that are being developed to predict Aβ status can circumvent these limitations [18]. Several studies have revealed that the plasma Aβ_1-42_ (Aβ42) to Aβ_1-40_ (Aβ40) ratio (Aβ42/Aβ40) has the potential to predict Aβ status as defined by amyloid PET or CSF testing [19–22]. Some of these assays use immunoprecipitation mass spectrometry (IP-MS), which enables the highly specific measurement of target analytes [23–25]. A recent study showed that IP-MS assays perform better in predicting Aβ status than do immunoassays [26]. However, immunoassays are advantageous due to their higher throughput and greater accessibility.

Consequently, we developed highly specific plasma Aβ40 and Aβ42 immunoassays that show a significant correlation with IP-MS assays [27]. We used a fully automated immunoassay platform (HISCL series; Sysmex Corporation, Kobe, Japan), which is currently used in clinical settings. This platform uses a chemiluminescence enzyme methodology and is advantageous as compared to conventional enzyme-linked immunosorbent assays, given its robustness and rapid reaction time, requiring only 17 min to complete the measurements [28–30].

Here, we describe the clinical performance of our plasma Aβ40 and Aβ42 immunoassays. The accuracy of the plasma Aβ42/Aβ40 ratio for predicting Aβ status defined by amyloid PET was examined using two separate sample sets (discovery and validation studies) from specimens collected during screening for the beta-site APP-cleaving enzyme inhibitor elenbecestat Phase 3 program (MissionAD1 NCT02956486, MissionAD2 NCT03036280) targeting patients with early AD.

## Materials and methods

### Participants and samples

Plasma samples were retrospectively obtained from the elenbecestat Phase 3 program, which consisted of two global multicenter clinical trials (MissionAD1 NCT02956486, MissionAD2 NCT03036280). In these trials, participants were clinically diagnosed with early AD (mild cognitive impairment [MCI] due to AD and mild AD) according to the core clinical criteria established by the NIA-AA2011 diagnostic guidelines [31, 32]. The participants satisfying the core clinical criteria were assessed for their Aβ status by amyloid PET (visual reading of florbetapir, florbetaben, or flutemetamol PET) or CSF test in the screening phase of the elenbecestat Phase 3 program. *APOE* ε4 status was defined as positive if participants had at least one ε4 allele based on real-time polymerase chain reaction results and as negative if they did not have an ε4 allele.

Of these participants, we randomly selected 200 plasma samples for the discovery and validation studies as two different sample sets from participants who underwent amyloid PET to confirm Aβ status. Plasma samples were collected during the screening phase of the trials. The samples were selected to yield approximately 50% positive prevalence of amyloid PET results defined by PET visual read, under the conditions of having both 25% of Japanese participants and 20% of participants with mild AD in each sample set.

All studies were conducted in accordance with the Declaration of Helsinki and after approval of the local ethics committees of the participating centers. All participants (or their legal representatives) provided written informed consent prior to enrolling in MissionAD.

### Plasma collection and analysis

Blood samples were acquired by completely filling a 6-mL plastic K2 EDTA tube (Becton, Dickinson and Company, Franklin Lakes, NJ, USA). Within 30 min of blood collection, samples were centrifuged at 2000 × *g* for 10 min. After centrifugation, plasma (0.5 mL) was transferred into 2.0-mL Protein Lobind tubes (Eppendorf, Hamburg, Germany). Within 30 min of plasma separation, the plasma samples were transferred to a freezer (below − 70 °C).

The plasma Aβ40 and Aβ42 levels were measured using the HISCL series. The Aβ assays required 30 μL of plasma, and measurements were completed within 17 min per assay. These assay procedures have been previously described [27].

To evaluate the clinical performance of our immunoassay, plasma Aβ42/Aβ40 was used. A cutoff value was determined by maximizing the Youden Index in the discovery sample set. The sensitivity, specificity, positive-predictive value (PPV), and negative-predictive value (NPV) were calculated in both the discovery and validation studies using the same cutoff value.

### Amyloid PET

Three amyloid PET probes (florbetaben, florbetapir, and flutemetamol) were used, depending on their availability. The brain Aβ status was centrally assessed by a radiologist in a blinded manner to cognitive status, using the visual read method developed for each PET probe. All scans were independently analyzed by readers trained with respect to the guidelines established by the manufacturer. Centiloids (CL) were calculated by Bioclinica (imaging CRO) using their own CL imaging pipeline, based on the methods described in Klunk et al. (2015) [33, 34]. For the exploratory analysis, a CL of 32.21 was used as the cutoff value for Aβ-positivity, which was assessed based on receiver operating characteristic (ROC) curve analysis and determination of the Youden Index using visual reads as the standard for each of the three amyloid PET probes in the elenbecestat Phase 3 program (*n* = 3,492) [35].

### Statistical analyses

Statistical analyses were conducted using StatFlex version 7.0 software (Artech Co., Ltd., Osaka, Japan) and Analyse-it version 5.90 software (Analyse-it Software, Ltd.; https://analyse-it.com/). Differences between the discovery and validation sample sets were evaluated using the Student’s *t*-test for continuous measures and the *χ*^2^ test for categorical variables. To assess the overall performance in predicting amyloid PET status, the area under the ROC curve (AUC) was obtained by performing ROC analysis using logistic regression. The DeLong method was used to calculate 95% confidence intervals (CI) and to compare the difference in AUCs [36]. The 95% CIs of sensitivity, specificity, PPV, and NPV were calculated based on Clopper‒Pearson exact CIs. Correlation analysis was performed using Spearman rank correlation. For all analyses, *P* values < 0.05 were considered significant.

## Results

### Participants and samples

We analyzed 197 and 200 samples for the discovery and validation studies, respectively, because three samples in the discovery study were excluded due to measurement errors. Two samples were excluded due to an insufficient amount of plasma samples, and one sample was excluded due to severe hemolysis. Demographic characteristics of the discovery and validation studies are shown in Table 1. Except for the amyloid PET probe, none of the demographics revealed statistically significant differences between the discovery and validation sample sets. Each sample set contained 25% Japanese individuals (50 of 197 in the discovery study and 50 of 200 in the validation study) and 20% participants with mild AD (40 of 197 in the discovery study and 40 of 200 in the validation study). The time interval between amyloid PET and plasma collection for the discovery and validation studies corresponded to a median of 41 days (range, 10–321 days) and 36 days (range, 11–230 days), respectively.

**Table 1:** Participant demographics

	**Discovery study (*n* = 197)**	**Validation study (*n* = 200)**	***P* value**
Age (years), mean ± SD	71.1 ± 8.5	70.8 ± 7.9	NS
Sex, female/male	112/85	99/101	NS
Race, White/Japanese/Other	130/50/17	136/50/14	NS
*APOE* ε4 status, − / + /NA	123/71/3	114/86/0	NS
Amyloid PET status, − / +	97/100	100/100	NS
Amyloid PET probe, FBB/FBP/FMM/NA	124/27/46/0	124/22/45/9	0.023
MMSE, mean ± SD	26.4 ± 1.8	26.5 ± 1.8	NS
CDR-SB, mean ± SD	2.4 ± 1.0	2.5 ± 1.0	NS
Centiloid unit, mean ± SD	38.4 ± 50.0 (*n* = 180)	41.4 ± 48.9 (*n* = 191)	NS
Clinical disease staging, MCI due to AD/mild AD	157/40	160/40	NS

Significant differences between the discovery and validation studies were evaluated via the Student’s *t*-test for continuous measures and the *χ*.^2^ test for categorical variables

*Abbreviations*: *PET* Positron emission tomography, *SD* Standard deviation, *NS* Not significant, *APOE* Apolipoprotein E, *NA* not available, *FBB* Florbetaben, *FBP* Florbetapir, *FMM* Flutemetamol, *MMSE* Mini-Mental State Examination, *CDR-SB* Clinical Dementia Rating-Sum of Boxes, *MCI* Mild cognitive impairment, *AD* Alzheimer’s disease

### Distribution of plasma Aβ levels across amyloid PET-positive and PET-negative groups

**Fig. 1:**
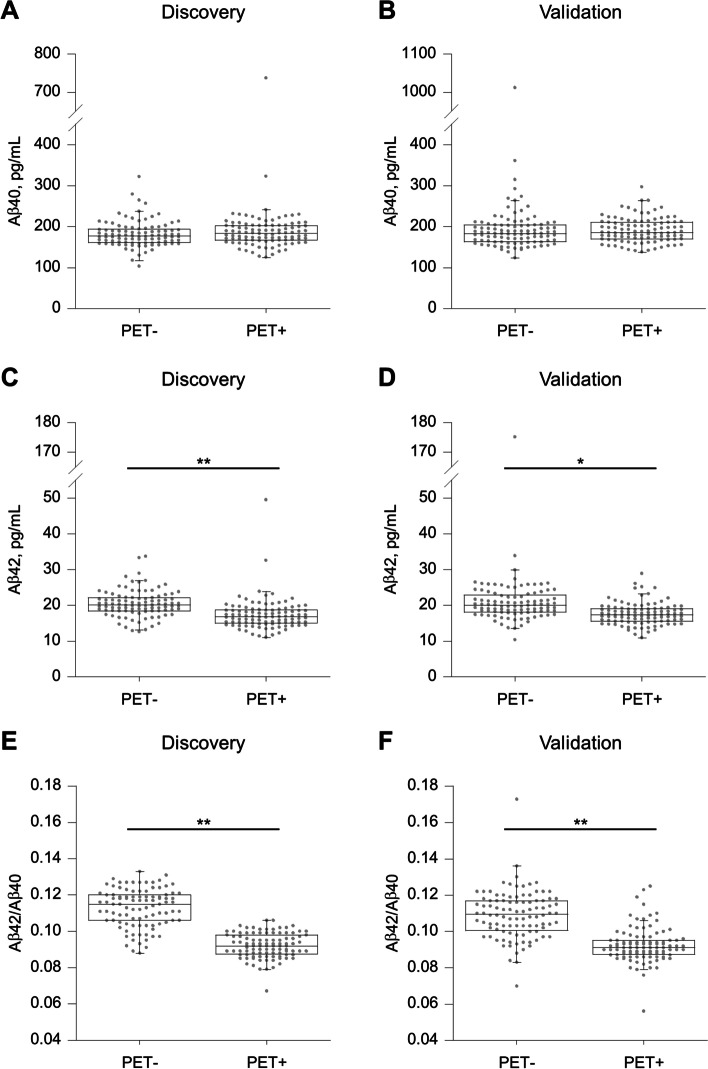
Distribution of plasma β-amyloid (Aβ) levels across amyloid positron emission tomography (PET)-positive and -negative groups. Plasma **A** Aβ40, **C** Aβ42, and **E** Aβ42/Aβ40 ratios in the discovery study. Plasma **B** Aβ40, **D** Aβ42, and **F** Aβ42/Aβ40 ratios in the validation study. Amyloid PET status was assessed by visual reads. The significance of the difference between groups was determined by using the Student’s *t* test. *P < 0.01, **P < 0.001

In both the discovery and validation studies, there was no statistically significant difference in the plasma Aβ40 levels between the amyloid PET-positive and PET-negative groups, which was confirmed by visual read (Fig. 1A, B). Conversely, plasma Aβ42 and Aβ42/Aβ40 values in the amyloid PET-positive group were statistically significantly lower than those in the amyloid PET-negative group (Fig. 1C‒F). The median values of Aβ42 in the amyloid PET-positive group were 16.2% (discovery study) and 13.4% (validation study) lower than those in the amyloid PET-negative group. The median values of the Aβ42/Aβ40 ratios in the amyloid PET-positive group were also 20.0% (discovery study) and 16.9% (validation study) lower than those in the amyloid PET-negative group.

### Performance of plasma Aβ levels in the prediction of brain Aβ status

**Fig. 2:**
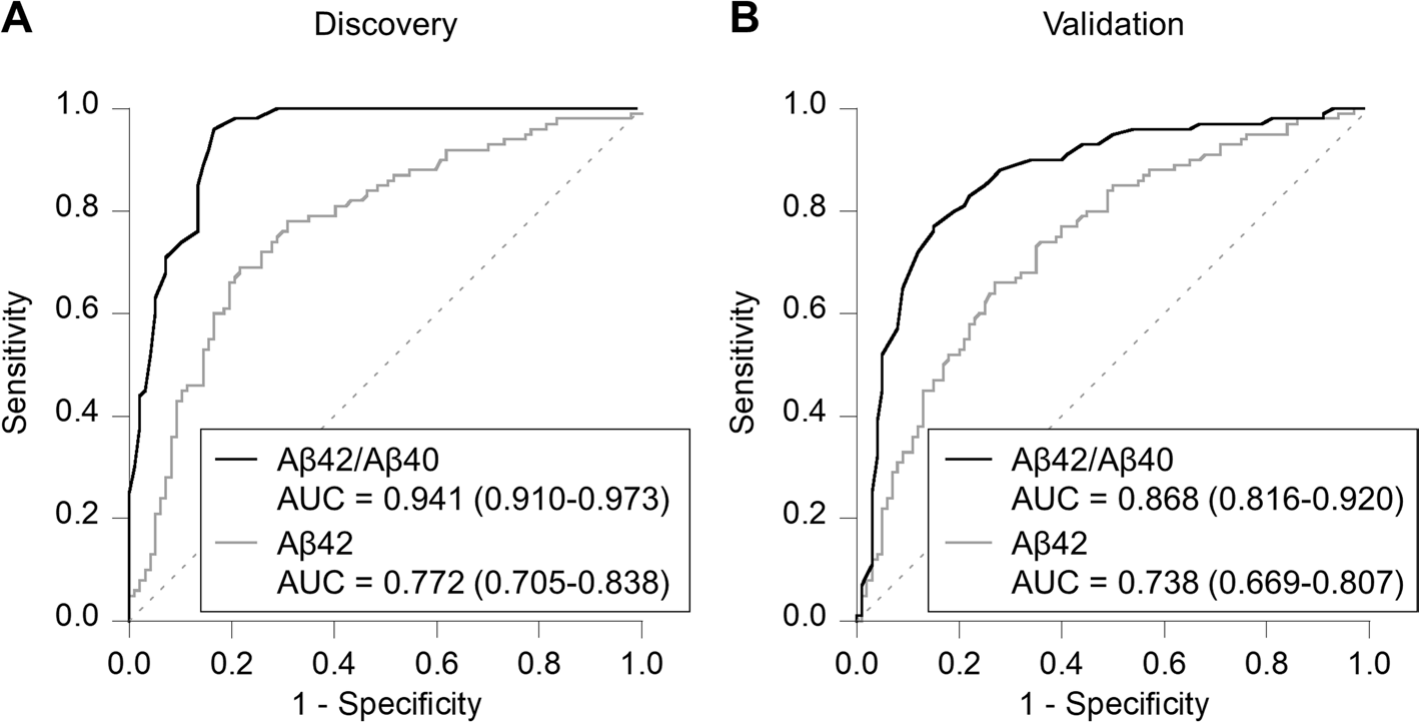
Performance of plasma Aβ levels in the prediction of amyloid PET status. Receiver operating characteristic (ROC) analyses for plasma Aβ42 and Aβ42/Aβ40 ratio in **A** discovery (*n* = 197) and **B** validation (*n* = 200) studies. The area under the curves (AUCs) are described with 95% confidence intervals. Amyloid PET status was assessed by visual reads

To assess the performance in predicting the brain Aβ status, as confirmed by amyloid PET visual read, ROC analyses were performed for plasma Aβ42 and Aβ42/Aβ40 in the discovery study (Fig. 2A). Plasma Aβ42 levels statistically significantly discriminated amyloid PET-positive from PET-negative groups, with an AUC of 0.772 (95% CI 0.705–0.838). Compared with that of the Aβ42 levels, the plasma Aβ42/Aβ40 had a statistically significantly better ability to predict brain Aβ status, with an AUC of 0.941 (95% CI 0.910–0.973).

We next assessed an Aβ42/Aβ40 cutoff value of 0.102, maximizing the Youden Index, for predicting the brain Aβ status. Using this cutoff value, the sensitivity, specificity, PPV, and NPV were calculated to be 96.0% (95% CI 90.1–98.9%), 83.5% (95% CI 74.6–90.3%), 85.7% (95% CI 77.8–91.6%), and 95.3% (95% CI 88.4–98.7%) in the discovery study (Table 2).

**Table 2:** Performance of plasma Aβ42/40 ratio for predicting amyloid PET status

		**% (95% CI)**			
Sample set	Cutoff	Sensitivity	Specificity	PPV	NPV
Discovery	0.102	96.0 (90.1–98.9)	83.5 (74.6–90.3)	85.7 (77.8–91.6)	95.3 (88.4–98.7)
Validation		88.0 (80.0–93.6)	72.0 (62.1–80.5)	75.9 (67.0–83.3)	85.7 (76.4–92.4)

The cutoff value was determined by maximizing the Youden Index. The 95% CIs were calculated based on the Clopper-Pearson exact CI

Abbreviations: *Aβ* β-amyloid, *PET* Positron emission tomography, *CI* Confidence interval, *PPV* Positive-predictive value, *NPV* Negative-predictive value

To verify the validity of the cutoff value, we evaluated the performance in predicting the brain Aβ status using the validation sample set. ROC analyses were also performed, and the AUCs of Aβ42 and Aβ42/Aβ40 were 0.738 (95% CI 0.669–0.807) and 0.868 (95% CI 0.816–0.920), respectively (Fig. 2B). The sensitivity, specificity, PPV, and NPV using the cutoff value established in the discovery study were 88.0% (95% CI 80.0–93.6%), 72.0% (95% CI 62.1–80.5%), 75.9% (95% CI 67.0–83.3%), and 85.7% (95% CI 76.4–92.4%), respectively (Table 2).

We also assessed the additional predictive power of *APOE* ε4 status on the plasma Aβ42/Aβ40 levels in predicting brain Aβ status. In this analysis, three samples from the discovery study were excluded due to the lack of *APOE* genotype information. We used 194 samples from the discovery study and 200 from the validation study to construct a logistic regression model to predict the amyloid PET status by adding the *APOE* ε4 status to plasma Aβ42/Aβ40 as predictors (Table S1). The AUCs were 0.950 (95% CI 0.921–0.980) in the discovery and 0.868 (95% CI 0.816–0.920) in the validation sample sets; however, these AUCs were not statistically significantly different compared to the AUCs for plasma Aβ42/Aβ40 alone, as calculated using the same sample sets (*n* = 194 for the discovery study and *n* = 200 for the validation study) (Fig. S1).

### Correlation between plasma Aβ42/Aβ40 and amyloid PET in Centiloid units

The Centiloid method has previously been used to standardize amyloid PET results. This method addresses differences that include amyloid PET probe characteristics, result-acquisition time, PET scanners, and imaging processing pipelines, enabling comparison across different PET probes and scanning sites. Thus, we calculated the CL and evaluated the performance of plasma Aβ42/Aβ40 to predict brain Aβ status, as determined by using a cutoff of 32.21 CL. In this analysis, 17 samples in the discovery study and nine in the validation study were excluded due to a lack of SUVr information. ROC analyses were performed in both discovery and validation studies. The AUCs of Aβ42/Aβ40 were 0.932 (95% CI 0.896–0.969) and 0.922 (95% CI 0.883–0.961) in the discovery and validation studies, respectively (Fig. S2).

**Fig. 3:**
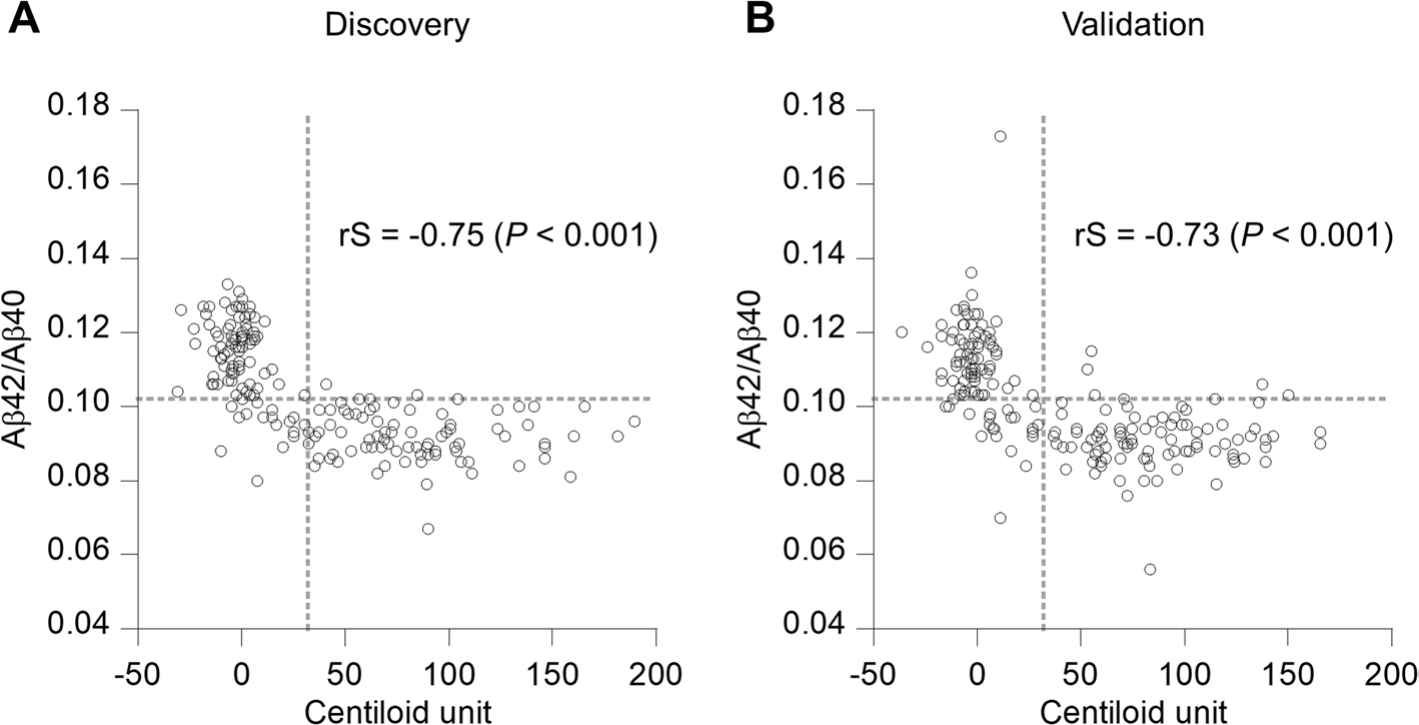
Correlation between plasma Aβ42/Aβ40 ratio and Centiloid unit. The Spearman rank correlation coefficient (rS) was calculated to assess the correlation in **A** discovery (*n* = 180) and **B** validation (*n* = 191) studies. Gray dashed line indicates the cutoff value for the Centiloid unit and the plasma Aβ42/Aβ40 ratio

The correlation between plasma Aβ42/Aβ40 levels and CL was also evaluated (Fig. 3). In both studies, plasma Aβ42/Aβ40 and CL were significantly correlated with a Spearman rank correlation coefficient of − 0.75 for the discovery and − 0.73 for the validation sample sets (*P* < 0.001).

In the discovery study, 97.5% (79 of 81 samples) of amyloid PET-positive samples, as determined by CL (CL-positive), were also plasma Aβ42/Aβ40-positive. Only two CL-positive samples (2.5%) were categorized as negative for plasma Aβ42/Aβ40. Regarding the amyloid PET-negative samples, as determined by CL (CL-negative), 80.8% (80 of 99 samples) were concordant with plasma Aβ42/Aβ40 results. The remaining 19.2% (19 of 99 samples) were plasma Aβ42/Aβ40-positive. Similar trends were identified in the validation study using the same cutoff values. Overall, 94.5% of CL-positive (86 of 91) and 78.0% of CL-negative (78 of 100) samples were concordant with plasma Aβ42/Aβ40 results. However, 5.5% of CL-positive (5 of 91) and 22% of CL-negative (22 of 100) samples had discordant plasma Aβ42/Aβ40 findings.

## Discussion

In this study, we described the clinical performance of fully automated immunoassays using the HISCL series to measure the plasma Aβ42/Aβ40 ratio. Our immunoassay system achieved high accuracy in predicting brain Aβ status, defined via visual reads of amyloid PETs, with AUCs of 0.941 (discovery study) and 0.868 (validation study). Our immunoassay system achieved 96.0% sensitivity and 83.5% specificity in the discovery study and 88.0% sensitivity and 72.0% specificity in the validation study. This indicated that the plasma Aβ42/Aβ40 ratio determined using our immunoassay demonstrated a high predictive performance for detecting the brain Aβ status.

Several reports have shown that a decrease in the CSF Aβ42/Aβ40 ratio is related to Aβ pathology in the brain, suggesting that the CSF Aβ42/Aβ40 ratio is a promising biomarker for Aβ pathology [19, 37, 38]. While CSF testing may be more accessible and economical than PET, it has the disadvantage of requiring a restrictive lumbar puncture, which is an invasive procedure that requires suitable staff, training, and facilities and which has some potential for complications [16, 17]. Previous reports have demonstrated that CSF Aβ42/Aβ40 ratios predicted the brain Aβ status determined by visual reads of amyloid PET with AUCs of 0.92–0.95, sensitivities of 91–99%, and specificities of 82–89% [39, 40]. Consistent with these previously reported CSF assays, plasma Aβ42/Aβ40 ratio in the current study predicted the brain Aβ status. Moreover, compared to CSF-based biomarkers, blood-based biomarkers are more easily assessable, given that blood sample collection is minimally invasive.

IP-MS-based assays for plasma Aβ40 and Aβ42 have shown that a decrease in the plasma Aβ42/Aβ40 ratio has high accuracy in predicting brain Aβ pathology [21, 22]. Given the high and accelerating incidence of AD and anticipated growth of DMTs, plasma-based immunoassays could provide an accessible and higher throughput biomarker for assessing the brain Aβ status. Usually, immunoassays have difficulties in demonstrating sufficient specificity, particularly for the measurement of proteins that have several types of fragments, such as Aβ, because the specificity of the immunoassay depends on the specificity of the antibodies. We confirmed the specificity of our immunoassays by evaluating their correlation with IP-MS assays [27] and found that our assay accurately measured the Aβ42 and Aβ40 levels in plasma. The findings provide proof of principle that our immune-based plasma Aβ42/Aβ40 assay accurately distinguishes brain Aβ-positive from Aβ-negative individuals, as determined by visual reads of amyloid PET, with AUCs of 0.941 and 0.868 in the discovery and validation studies, respectively.

*APOE* ε4 is widely considered a major risk factor for the development of AD [41]. Previous studies have reported that *APOE* ε4 status increases the performance of plasma Aβ42/Aβ40 to predict brain Aβ status [20, 42]. In this study, we observed a slight numerical increase in the performance of the plasma Aβ42/Aβ40 ratio on incorporating the *APOE* ε4 status; however, this was not significantly different, suggesting that our assay may have high performance, possibly even without including *APOE* ε4 status.

The Centiloid method, a standardized quantitative measurement scale, enables comparisons across different amyloid PET probes and sites on the same scale. In light of these advantages, other research groups recently calculated the CL and assessed the performance of blood-based biomarkers for assessing amyloid PET status [22, 43–45]. In this study, we measured samples obtained from multiple sites using three different PET probes. We calculated the CL and evaluated the performance of plasma Aβ42/Aβ40 to predict amyloid PET status as determined by the CL cutoff. Our plasma Aβ42/Aβ40 ratio had high accuracy in predicting amyloid PET status as determined by CL cutoff, with AUCs of 0.932 and 0.922 in the discovery and validation studies, respectively. We also confirmed a significant correlation between plasma Aβ42/Aβ40 and CL. The amyloid PET CL value reflects the total amount of Aβ in the brain, indicating that our plasma assays may reflect this amount.

However, we observed a number of disagreements between the plasma Aβ42/Aβ40 and CL results. These disagreements were mostly observed in the bottom-left area of Fig. 3, corresponding to the plasma Aβ42/Aβ40-positive and CL-negative cases. These disagreements have been reported by other groups and represent participants who have an increased risk of conversion to amyloid PET-positivity [22], potentially highlighting that our plasma assays may detect Aβ pathology earlier than would PET imaging.

In light of the first potential disease-modifying treatment for AD becoming available in the USA, confirming Aβ pathology in patients is becoming increasingly important. Given that the current targets of these drugs are patients with early AD, our immunoassay system could enable the confirmation of brain Aβ status and may facilitate access to treatment for appropriate patients.

### Limitations

This study was limited in that, although the two sample sets for the discovery and validation studies were completely independent, they were derived from the same clinical program. Moreover, the discovery and validation studies were conducted in a population with a clinical diagnosis of MCI due to AD or mild AD. These results should be confirmed in different cohorts with more diverse populations, including cognitively unimpaired subjects, racially diverse populations, participants with other neurodegenerative diseases, and real-world, non-clinical trial populations.

## Conclusion

In conclusion, we developed a fully automated plasma Aβ immunoassay that can predict brain Aβ status with high accuracy. Since aducanumab has already been approved in the US, and since Biologics License Applications submissions of other potential anti-Aβ antibodies have already been initiated, more widely available tests to confirm brain Aβ deposition that are less expensive, less invasive, and easily accessible would reduce the burden on patients and health care providers. Further clinical research using different samples is required. However, this assay may have the potential to fundamentally contribute to the pathophysiological diagnosis of AD in clinical practice.

## Supplementary Information

**Additional file 1: Table S1.** Logistic regression model of plasma Aβ42/Aβ40 ratio combined with APOE ε4 status in the prediction of amyloid PET status. **Figure S1.** Logistic regression model of plasma Aβ42/Aβ40 ratio combined with APOE ε4 status in the prediction of amyloid PET status. **Figure S2.** Performance of plasma Aβ levels in the prediction of amyloid PET status determined by Centiloid unit.

## Data Availability

The datasets generated and/or analyzed during the current study are not publicly available due to ethical issues but are available from the corresponding author on reasonable request.

## Abbreviations

AD: Alzheimer’s disease
AUC: Area under the ROC curve
CI: Confidence interval
DMT: Disease-modifying therapies
MCI: Mild cognitive impairment
NPV: Negative-predictive value
PET: Positron emission tomography
PPV: Positive-predictive value
ROC: Receiver operating characteristic
