# Supplementary material for: Fully automated and highly specific plasma β-amyloid immunoassays predict β-amyloid status defined by amyloid positron emission tomography with high accuracy
Source: Alzheimers Res Ther. 2022 Jun 23;14:86. doi: 10.1186/s13195-022-01029-0 (PMC9219197; doi:10.1186/s13195-022-01029-0)
Supplement: Supplementary file 1 — Additional file 1: Table S1. Logistic regression model of plasma Aβ42/Aβ40 ratio combined with APOE ε4 status in the prediction of amyloid PET status. Figure S1. Logistic regression model of plasma Aβ42/Aβ40 ratio combined with APOE ε4 status in the prediction of amyloid PET status. Figure S2. Performance of plasma Aβ levels in the prediction of amyloid PET status determined by Centiloid unit. [file 13195_2022_1029_MOESM1_ESM.pdf]

Supplemental Material

Yamashita K, Miura M, Watanabe S, et al. Fully automated and highly specific plasma β-amyloid immunoassays predict β-amyloid status defined by amyloid PET with high accuracy

Table S1. Logistic regression model of plasma Aβ42/Aβ40 ratio combined with APOE ε4 status in the prediction of amyloid PET status.

Figure S1. Logistic regression model of plasma Aβ42/Aβ40 ratio combined with APOE ε4 status in the prediction of amyloid PET status.

Figure S2. Performance of plasma Aβ levels in the prediction of amyloid PET status determined by Centiloid unit.

Table S1. Logistic regression model of plasma Aβ42/Aβ40 ratio combined with *APOE* ε4 status in the prediction of amyloid PET status

	Discovery			Validation		
	Estimate	SE	*P* value	Estimate	SE	*P* value
Intercept	25.05	3.889		12.71	2.031	
Aβ42/Aβ40	-253.4	39.16	< 0.001	-129.3	19.94	< 0.001
APOE ε4 status	1.446	0.5350	< 0.01	0.4594	0.3789	0.2253

Amyloid PET status was predicted by plasma Aβ42/Aβ40 ratio and *APOE* ε4 status (*APOE* ε4 positive or negative) as predictors in logistic regression model. Amyloid PET status was determined by visual read method.

Abbreviations: Aβ, β-amyloid; APOE, Apolipoprotein E; SE, standard error; PET, Positron Emission Tomography

Figure S1

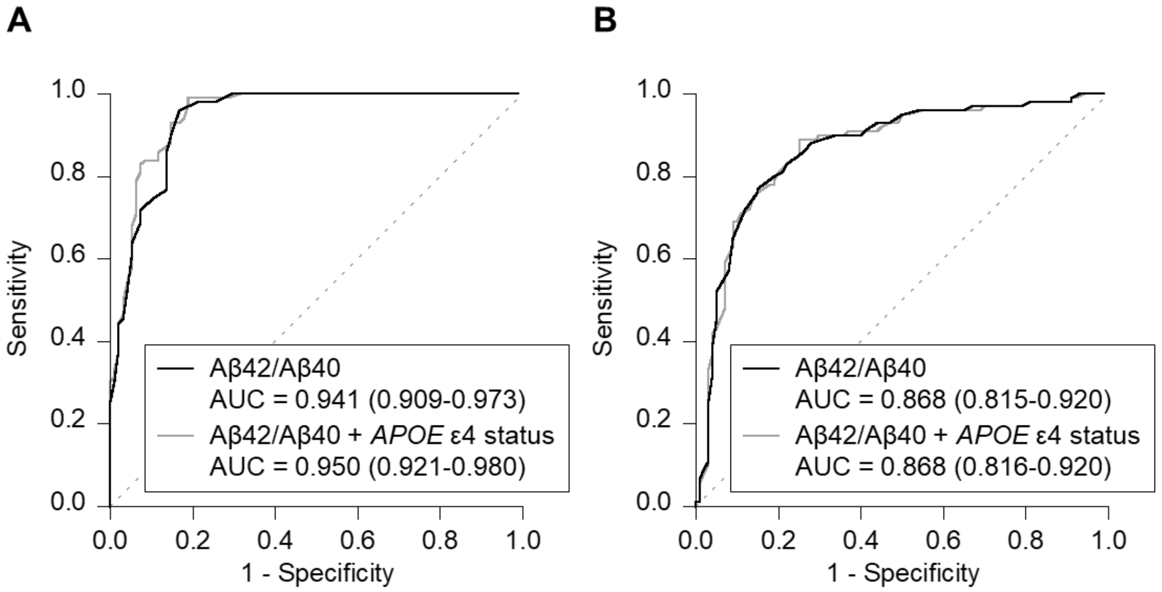

Fugure S1. Logistic regression model of plasma Aβ42/Aβ40 ratio combined with *APOE* ε4 status in the prediction of amyloid PET status. Receiver operating characteristic (ROC) analyses for Aβ42/Aβ40 ratio and the model including *APOE* ε4 status in (A) discovery (n = 194) and (B) validation (n = 200) studies. The area under the curves (AUCs) are described with 95% confidence intervals. Amyloid PET status was determined by visual reads.

Figure S2

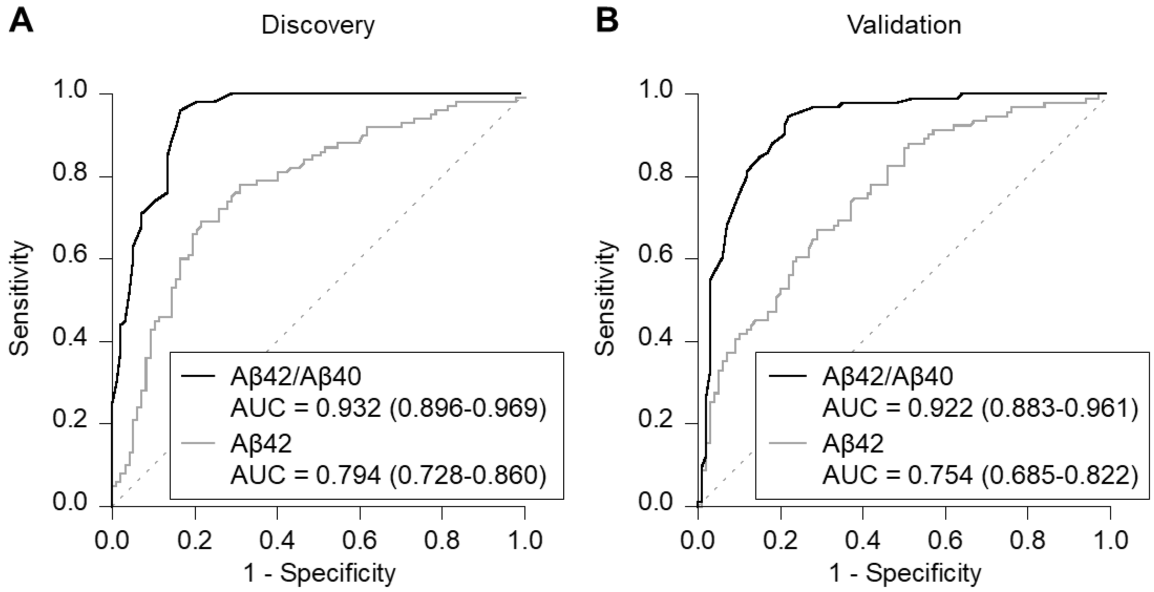

Figure S2. Performance of plasma Aβ levels in the prediction of amyloid PET status determined by Centiloid unit. Receiver operating characteristic (ROC) analyses for plasma Aβ42 and Aβ42/Aβ40 ratio in (A) discovery (n = 180) and (B) validation (n = 191) studies. The area under the curves (AUCs) are described with 95% confidence intervals. Amyloid PET status was determined by Centiloid unit.
